# Damage-associated responses of the host contribute to defence against cyst nematodes but not root-knot nematodes

**DOI:** 10.1093/jxb/erx374

**Published:** 2017-10-19

**Authors:** Syed Jehangir Shah, Muhammad Shahzad Anjam, Badou Mendy, Muhammad Arslan Anwer, Samer S Habash, Jose L Lozano-Torres, Florian M W Grundler, Shahid Siddique

**Affiliations:** 1 Rheinische Friedrich-Wilhelms-University of Bonn, INRES – Molecular Phytomedicine, Germany; 2 Laboratory of Nematology, Wageningen University, Wageningen, The Netherlands

**Keywords:** Damage-associated molecular patterns (DAMPs), glucosinolate, nematode, oligogalacturonide (OG), pattern-triggered immunity (PTI), plant-parasitic nematodes, polygalacturonase (PG), polygalacturonase-inhibiting protein (PGIP)

## Abstract

When nematodes invade and subsequently migrate within plant roots, they generate cell wall fragments (in the form of oligogalacturonides; OGs) that can act as damage-associated molecular patterns and activate host defence responses. However, the molecular mechanisms mediating damage responses in plant–nematode interactions remain unexplored. Here, we characterized the role of a group of cell wall receptor proteins in Arabidopsis, designated as polygalacturonase-inhibiting proteins (PGIPs), during infection with the cyst nematode *Heterodera schachtii* and the root-knot nematode *Meloidogyne incognita*. PGIPs are encoded by a family of two genes in Arabidopsis, and are involved in the formation of active OG elicitors. Our results show that *PGIP* gene expression is strongly induced in response to cyst nematode invasion of roots. Analyses of loss-of-function mutants and overexpression lines revealed that *PGIP1* expression attenuates infection of host roots by cyst nematodes, but not root-knot nematodes. The PGIP1-mediated attenuation of cyst nematode infection involves the activation of plant camalexin and indole-glucosinolate pathways. These combined results provide new insights into the molecular mechanisms underlying plant damage perception and response pathways during infection by cyst and root-knot nematodes, and establishes the function of *PGIP* in plant resistance to cyst nematodes.

## Teaser

Expression of polygalacturonase-inhibiting protein (PGIP) genes and production of oligogalacturonides (OGs) form an important component of plant basal resistance against cyst nematodes, but not root-knot nematodes.

## Introduction

Plant-parasitic nematodes attack almost all major crops throughout the world, causing damage that has been estimated at >US$100 billion per year (Nicol *et al.*, 2011). The ~4100 known species of plant-parasitic nematodes (Decraemer and Hunt, 2006) display a wide variety of parasitic strategies, including simple migratory endoparasites that live in soil and feed on different tissue layers, and more complex migratory endoparasites that move continuously as they feed, thereby causing extensive necrosis of the infected tissues. However, the most complex and economically important is a group of sedentary endoparasites that includes cyst nematodes (CNs; *Globodera* spp. and *Heterodera* spp.) and root-knot nematodes (RKNs; *Meloidogyne* spp.). Infective-stage CN and RKN juveniles (J2) invade the plant root near the tip and move through different tissue layers to reach the vascular cylinder. Once inside the root, RKN J2s move intercellularly, whereas CN J2s move intracellularly, causing more damage to the host tissues. After reaching the vascular cylinder, CNs induce the formation of a syncytium, whereas RKNs induce the formation of 5–7 giant cells. Both the syncytium and giant cells are hypermetabolic sink tissues, and serve as the sole source of nutrients for growing nematodes throughout their entire life cycle (Kyndt *et al.*, 2013; Siddique and Grundler, 2015). In the case of RKNs, the development of giant cells is accompanied by hypertrophy and hyperplasia of neighbouring tissues, leading to the formation of typical knot-like galls in roots.

The first barrier encountered by nematodes during root invasion is the cell wall. Nematodes utilize two strategies to penetrate the plant cell wall: a stylet is used to pierce through the wall, and an array of cell wall-degrading enzymes is secreted to disrupt wall rigidity, including pectate lyase (de Boer *et al.*, 2002; Vanholme *et al.*, 2007), endo-β-1, 4-glucanase (Smant *et al.*, 1998; de Boer *et al.*, 1999), and polygalacturonase (PG) (Jaubert *et al.*, 2002). PGs are key enzymes that cleave the α1–4 linkage between the d-galacturonic acid residues of homogalacturonan (Kalunke *et al.*, 2015; Rahman and Joslyn, 1953*b*; Themmen *et al.*, 1982). PGs are well characterized in fungi, bacteria, and insects, and their action on the outer plant cell wall is essential for further wall degradation by other wall-degrading enzymes (Rahman and Joslyn, 1953a, b; Kester and Visser, 1990). Several fungi secrete PGs, including *Aspergillus flavus* (Whitehead *et al.*, 1995), *Botrytis cinerea* (Cabanne and Doneche, 2002; Favaron *et al.*, 1992), *Aspergillus niger* (Maldonado and de Saad, 1998), *Claviceps purpurea* (Oeser *et al.*, 2002), and *Sclerotinia sclerotiorum* (Reymond-Cotton *et al.*, 1996). A number of bacteria also produce PGs, including *Agrobacterium tumefaciens* (Rodriguezpalenzuela *et al*., 1991), *Ralstonia solanacearum* (Huang and Allen, 2000), and *Bacillus polymyxa* (Nagel and Vaughn, 1961). Similarly, the salivary glands of some insect species that feed on plants produce PGs, which help them feed on host tissues (Strong and Kruitwagen, 1968; Laurema *et al.*, 1985; Celorio-Mancera *et al.*, 2008, 2009). As stated above, nematodes also secrete PGs. In fact, the first PG of animal origin was isolated from the RKN *Meloidogyne incognita*, where it has been suggested to have a role in parasitism (Jaubert *et al.*, 2002). In addition, the transcriptome of the beet cyst nematode (BCN), *Heterodera schachtii*, was recently described to encode a PG (Fosu-Nyarko *et al.*, 2016).

Plant cell walls can inhibit microbial PG activity via a leucine-rich repeat defence protein called PG-inhibiting protein (PGIP), which attenuates pectin degradation. The crystal structure of PGIP contains a central leucine-rich repeat domain with 10 imperfect repeating units, each derived from 24 amino acid residues. Most leucine-rich repeat proteins have one β-sheet connected with a helix on the convex side or β-turns (Di Matteo *et al.*, 2003). In contrast, the leucine-rich repeat domain in PGIP is organized to form two β-sheets; sheet B1 occupies the concave inner side of the molecule and contains amino acid residues that are crucial for interactions with PGs (Di Matteo *et al.*, 2003). The association of PGIP with PG inhibits PG-mediated cell wall degradation and generates oligogalacturonides (OGs) with elicitor activity (Bishop *et al.*, 1981; Hahn *et al.*, 1981; Nothnagel *et al.*, 1983; Benedetti *et al.*, 2015). These OGs have a degree of polymerization between 10 and 15 (Cote and Hahn, 1994), and they activate defence responses such as the reactive oxygen species (ROS) burst (Galletti *et al.*, 2008), callose deposition (Bellincampi *et al.*, 2000), phytoalexins (Davis *et al.*, 1986), and nitric oxide (Rasul *et al.*, 2012).

The importance of PGIPs in nematode infection is supported by a study in pea (*Pisum sativum* L.) where *PsPGIP1* has been shown to be differentially expressed in susceptible and resistant genotypes in response to *Heterodera goettingiana* infection (Veronico *et al.*, 2011). *In situ* hybridization analysis confirmed that *PsPGIP1* is localized specifically in the syncytium of a resistant pea genotype, suggesting that *PsPGIP1* disrupts syncytium development inside the host root (Veronico *et al.*, 2011). Further progress in this field requires a detailed analysis of the roles of PG, PGIP, and OG in plant–nematode interactions (Holbein *et al.*, 2016). Here, we investigate the role of PGIPs in Arabidopsis during infection with the BCN *H. schachtii* and the RKN *M. incognita*. We found that *PGIP1*-mediated defence responses form an important component of host basal resistance to CNs but not to RKNs.

## Materials and methods

### Plant growth conditions and nematode infection assays

Arabidopsis plants were grown in either Knop medium (for BCN infection) or Murashige and Skoog (MS) medium (for RKN infection) as described previously (Siddique *et al.*, 2015). The T-DNA insertion mutants were ordered from the Nottingham stock centre (*pgip1-1*, SALK_001662.33.10.x. *pgip1-2*, GK-092G09-012001, *pgip2-1*, and GK-717A02-025309). Salk lines were genotyped (Supplementary Fig. S1 at *JXB* online) using primers listed in Supplementary Table S1. GK lines were screened for homozygosity through sulfadiazine resistance. The homozygous T-DNA insertion mutants were checked for lack of expression (Supplementary Fig. S2) using the primers listed in Supplementary Table S1. Twelve-day-old plants were infected with surface-sterilized 60–80 J2 individuals of BCN or RKN (*M. incognita*). For BCN, the average number of males and average number of females was counted at 12 days post-inoculation (dpi) (Siddique *et al.*, 2015). For RKN, the average number of galls was determined at 21 dpi. All infection assays for BCN and RKN were repeated a minimum of three times and each experiment consisted of 15–20 individual plants. The average area of syncytia and average female area were measured at 14 dpi as described previously (Siddique *et al.*, 2015). Approximately 30 syncytia and associated nematodes were measured for each experiment, and each experiment was repeated three times. To determine the average area of galls, ~30 galls were outlined and measured for each experiment, and each experiment was repeated three times

### Cloning and transformation of promoter::GUS lines

Promoter regions upstream of the start codons of *PGIP1* (1214 bp) and *PGIP2* (483 bp) as previously described by Ferrari *et al.* (2003) were amplified from genomic DNA using primers given in Supplementary Table S1 and cloned in a Gateway cloning vector, pDONR 207 (Invitrogen), according to the manufacturer’s instructions. The verified fragments were fused with the *β-glucuronidase* (*GUS*) gene in the expression vector pMDC162 (Curtis and Grossniklaus, 2003). These promoter::GUS constructs were introduced into *Agrobacterium tumefaciens* strain GV3101 for the transformation of 4- to 6-week-old Arabidopsis plants by the floral dip method (Clough and Bent, 1998). After drying of plants, seeds (T_0_) were harvested and sterilized before growing on Knop medium supplemented with 25 µg ml^−1^ hygromycin. Three independent homozygous plants were selected for further analysis. Homozygous lines were grown in Knop medium and infected with nematodes to analyse the GUS expression in a time-course analysis. The infected or uninfected roots were incubated with X-gluc for 12–14 h at 37 °C. After overnight incubation, the reaction was stopped and samples were washed with 70% ethanol. Staining was carried out at different time points for *H. schachtii* (1, 3, 5, and 10 dpi) and *M. incognita* (1, 3, 7, and 15 dpi). The stained syncytia and galls were photographed with a Leica DM4000 inverted microscope equipped with LAS software (Leica Microsystems) and fitted with an Olympus C-5050 digital camera.

### Quantitative RT–PCR

Arabidopsis plants were grown and infected with nematodes as described above. Root segments containing the infection zone were cut, and total RNA was extracted using an RNeasy Plant Mini Kit (Qiagen) following the manufacturer’s instructions. Contaminating DNA was digested with DNase1 using a DNA-*free*™ DNA Removal Kit (Ambion) and the RNA was used to synthesize cDNA using a High Capacity cDNA Reverse Transcription Kit (Applied Biosynthesis, Darmstadt, Germany) following the manufacturer’s instructions. Quantitative reverse transcription–PCR (qRT–PCR) was performed with the StepOne Plus Real-Time PCR System (Applied Biosystems) using the primers given in Supplementary Table S1. Each sample contained 10 μl of Fast SYBR Green qPCR Master Mix (Invitrogen), 2 mM MgCl_2_, 0.5 μl each of forward and reverse primers (10 μM), 2 μl of cDNA, and water in a 20 μl total reaction volume. *UBQ5* and *β-tubulin* was used as an endogenous control except for assays involving nematode feeding sites (galls and syncytia). For galls and syncytia, *18S* and *UBP22* were used as housekeeping genes as recommended previously (Hofmann and Grundler, 2007). cDNA was diluted 1:100 for 18S amplification. Data were analysed using Pfaffl’s method (Pfaffl, 2001). Data shown are an average of three independent experiments. Each experiment consisted of three technical replicates. Primer sequences used for qRT–PCR analysis along with their respective efficiencies are listed in Supplementary Table S1.

### Generation of overexpression and complementation lines

To overexpress *AtPGIP1* and *AtPGIP2*, full-length coding sequences of both genes were amplified from cDNA synthesized from RNA isolated from 12-day-old Arabidopsis plants. The primer pairs used to amplify the coding sequences from both genes are listed in Supplementary Table S1. The amplified PCR product was cloned into Gateway cloning vector pDONR207 (Invitrogen). The cloned fragments were verified through sequencing and transferred via Gateway recombination into the pMDC32 vector, where they were placed under the control of the double *Cauliflower mosaic virus* (CaMV) 35S promoter to engineer *AtPGIP1* and *AtPGIP2* overexpression. The verified constructs were introduced into *A. tumefaciens* strain GV3101, which was used for the transformation of 4- to 6-week-old Col-0 plants by the floral dip method (Clough and Bent, 1998). After drying of plants, seeds (T_0_) were harvested and sterilized before being sown on Knop medium supplemented with 25 µg ml^−1^ hygromycin. Transformants were selected to produce homozygous plants. At least two independent homozygous lines with the highest up-regulation were selected for further studies. Complemented lines of *pgip1* mutants were obtained by cloning a wild-type copy of the *PGIP1* gene under the control of the CaMV *35S* promoter using the Gateway cloning system as described above. Two homozygous complemented lines carrying an insertion of the wild-type gene were used in this study.

### Plant treatment with OGs

OGs with a degree of polymerization between 10 and 15 were purchased commercially (GAT114, Elicityl, France). Arabidopsis seeds were sterilized and grown in 6-well plates containing 5 ml of liquid Knop medium. After 9 d of germination, the medium was removed and 3 ml of fresh medium was added to the wells before adding 30 µl of OGs to a final concentration of 50 µg ml^−1^. After 24 h of treatment, the plants were gently placed in semi-solid Knop medium and allowed to recover from any stress for a few hours. Water-treated plants were used as a control and handled in the same manner. Afterwards, the OG- and water-treated plants were inoculated with 70–80 sterile J2s and evaluated for infection after 12–14 dpi as described above.

### Measurement of ROS

Apoplastic measurement of hydrogen peroxide in small root segments was carried out via a luminol-based detection method as previously described (Mendy *et al.*, 2017). Arabidopsis plants were grown in Knop medium for 2 weeks, after which uniform root pieces measuring ~0.2 cm were cut with a knife and placed in a 96-well plate with water for 24 h to reduce the wounding response. After overnight incubation, the water was removed and replaced with flg22 solution, and ROS was measured as described (Mendy *et al.*, 2017).

### Statistical procedures

Data were statistically analysed using SigmaPlot 12, applying *t*-test (*P*<0.05) for pairwise comparisons. For qPCR, statistical procedures were applied to ∆CT values as recommended previously (Livak and Schmittgen., 2001).

## Results

### *PGIP1* and *PGIP2* are induced by nematode infection

Arabidopsis plants contain a family of two *PGIP* genes designated as *PGIP1* and *PGIP2*. To assess the regulation of *PGIP* genes during different stages of nematode infection, we evaluated the expression of these genes in published transcriptomic data (Jammes *et al.*, 2005; Szakasits *et al.*, 2009; Barcala *et al.*, 2010; Mendy *et al.*, 2017). These analyses revealed that *PGIP1* expression increased during migratory (10 h post-inoculation, hpi) and sedentary stages of BCN infection with *H. schachtii* (Supplementary Table S2). In contrast, there were no significant differences in *PGIP1* and *PGIP2* expression levels in microarrays of root segments containing giant cells or galls infected with the RKN *M. javanica or M. incognita* (Jammes *et al.*, 2005; Barcala *et al.*, 2010; Cabrera *et al.*, 2014). However, a recent next-generation sequencing-based transcriptome profiling of Arabidopsis found that expression of both *PGIP1* and *PGIP2* is significantly up-regulated in galls (3, 5, and 7 dpi) induced by the RKN *M. incognita* (Yamaguchi *et al.*, 2017).

**Fig. 1.**
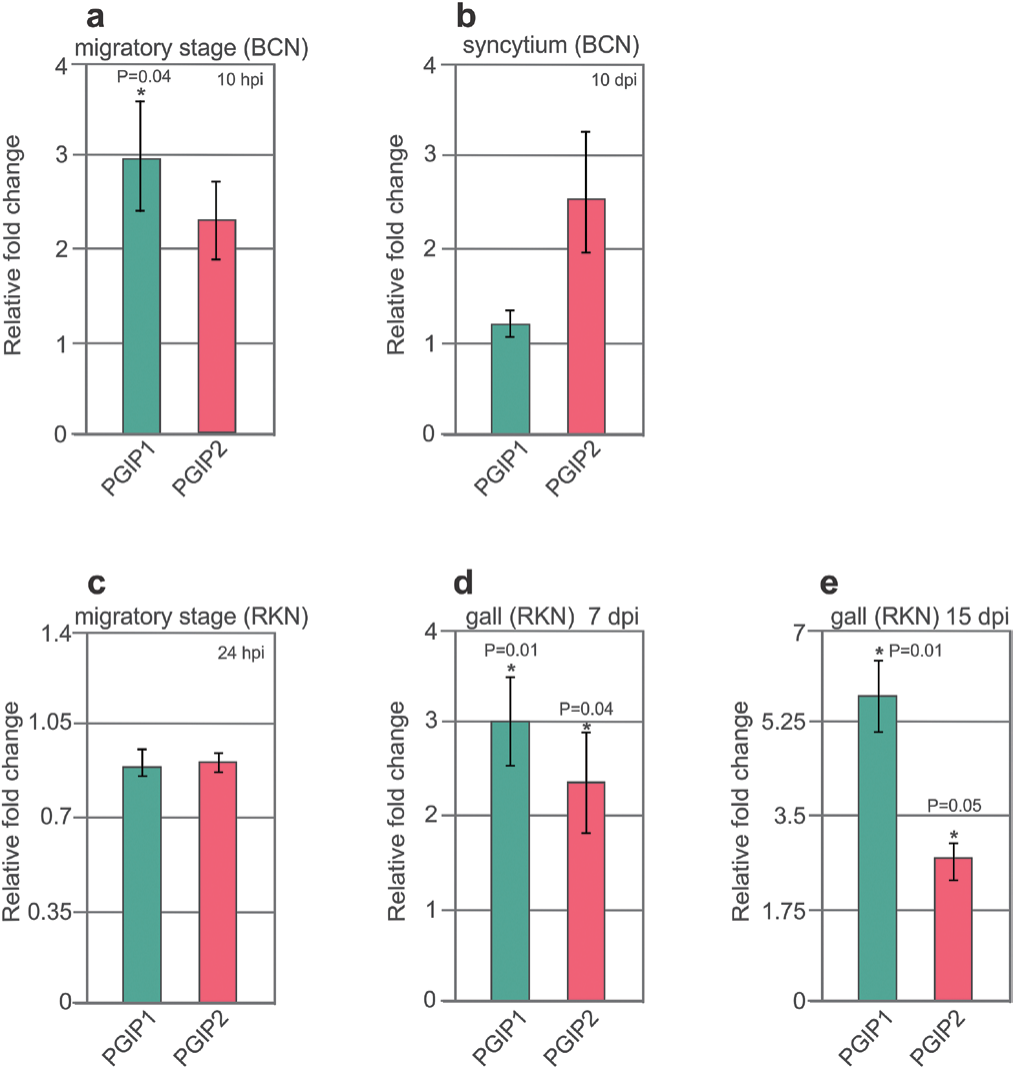
*PGIP* genes are activated in Arabidopsis upon nematode infection. Validation of changes in *PGIP* gene expression upon nematode infection via qRT–PCR. The values represent relative fold change in response to nematode infection with the value in uninfected control root set to 1. (a, c) *UBQ5* and *β-tubulin* were used as housekeeping genes to normalize the data. (b, d, e) *18S* and *UBP22* were used as housekeeping genes to normalize the data. (a–e) Data bars represent the mean ± SE for three independent experiments. Data were analysed using *t*-test (*P*<0.05). Asterisks represent statistically significant differences from uninfected control root.

We validated these microarray data using Arabidopsis plants that were grown *in vitro* and infected with BCNs or RKNs. RNA was extracted and analysed for the expression of *PGIP1* and *PGIP2* via qRT–PCR. For BCNs, infected root segments were sampled at 10 hpi (migratory stage; ~0.2 cm around the nematode head) or 10 dpi (sedentary stage). The results confirmed that *PGIP1* expression increases during the migratory stage at 10 hpi upon BCN infection (Fig. 1a), but we were unable to confirm *PGIP1* up-regulation during the sedentary stage at 10 dpi (Fig. 1b). For RKN, root segments were collected at 24 hpi (root tips; migratory stage), 7 dpi (sedentary stage), or 15 dpi (sedentary stage). We found no change in expression for *PGIP1* and *PGIP2* at the migratory stage with RKN (Fig. 1c), but the expression of both was increased during the sedentary stages at 7 dpi and 15 dpi (Fig. 1d, e).

**Fig. 2.**
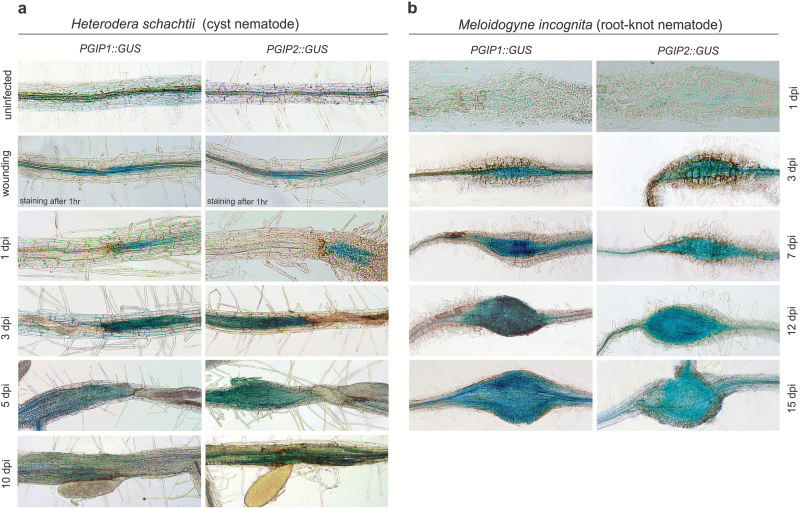
Activation of PGIP::GUS expression in Arabidopsis roots upon CN and RKN infection. (a) Expression of *PGIP1::GUS* and *PGIP2::GUS* in Arabidopsis roots upon wounding or *H. schachtii* infection at 1, 3, 5, and 10 dpi, respectively. Scale bar=200 µm. (b) Expression of *PGIP1::GUS* and *PGIP2::GUS* in Arabidopsis roots upon *M. incognita* infection at 1, 3, 7, 12, and 15 dpi, respectively. Scale bar=200 µm.

To determine the spatiotemporal expression patterns of *PGIP* genes during plant–nematode interactions, we transformed Arabidopsis with *PGIP1*::*GUS* or *PGIP2::GUS* constructs and generated 3–5 independent homozygous lines. Although *PGIP1* and *PGIP2* are induced by wounding in leaves, their expression patterns in roots have not been determined. Therefore, we wounded the roots of 10-day-old plants and performed GUS staining 1 h after wounding. We observed specific and strong GUS staining indicating *PGIP1* and *PGIP2* expression at and around the wounding sites (Fig. 2a). Next, we performed a time-course analysis of *PGIP* expression during BCN infection using the *PGIP* promoter::GUS fusions. The majority of root infection zones exhibited strong GUS staining at 1, 3, and 5 dpi, and no GUS staining was observed in uninfected root segments. The GUS staining intensity declined considerably at 10 dpi for both *PGIP1* and *PGIP2* (Fig. 2a). Next, we analysed the spatiotemporal expression patterns of *PGIP1::GUS* and *PGIP2::GUS* in response to infection with the RKN. No GUS staining was observed at 1 dpi for both *PGIP1* and *PGIP2*. In contrast, GUS-specific staining was observed at 3 dpi onward in galls induced by *M. incognita* (Fig. 2b). Taken together, we concluded that gene expression for both *PGIP1* and *PGIP2* is strongly induced during migratory stages of BCN infection but not during RKN migration.

### PGIP-mediated signalling is involved in cyst nematode infection

**Fig. 3.**
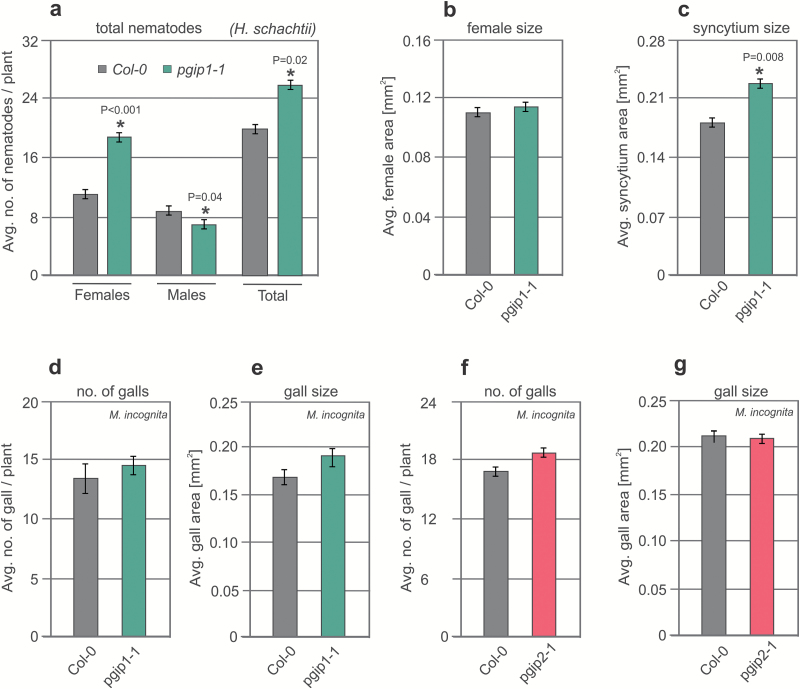
CN and RKN infection assays in PGIP1 and PGIP2 receptor mutant plants. (a) Average number of females and males per plant present in Col-0 *pgip1-1* mutant lines at 12 dpi. (b, c) Average sizes of female nematodes (b) and plant syncytia (c) in Col-0 and *pgip1-1* mutant lines at 14 dpi. (d, f) Average number of galls per plant present in Col-0, *pgip1-1* (d), and *pgip2-1* (f) mutant lines at 21 dpi. (e, g) Average area of galls per plant present in Col-0, *pgip1-1* (e), and *pgip2-1* (g) mutant lines at 21 dpi. (a–g) Bars represent the mean ± SE for three independent experiments. Data were analysed using *t*-test (*P*<0.05).

To explore the role of PGIPs in nematode infection, we characterized loss-of-function T-DNA insertion mutants for *PGIP1* (*pgip1-1* and *pgip1-2*) and *PGIP2* (*pgip2-1*) (Supplementary Figs S1, S2). Plants were grown *in vitro* for 12 d and then infected with J2s of BCN or RKN. For BCN, we counted the numbers of nematode females and males at 12 dpi, and the average syncytium size and average size of nematode females at 14 dpi. For RKN, we counted the number of galls and average area of galls at 21 dpi. After BCN infection, we observed a significant increase in the average number of females in both *PGIP1* mutants (*pgip1-1* and *pgip1-2*) compared with the Col-0 control (Fig. 3a; Supplementary Fig. S3a). Moreover, we also observed a significant increase in average syncytium size in *pgip1-1* and *pgip1-2* infected with BCN, but did not observe any significant differences in average female size (Fig. 3b, c; Supplementary Fig. S3b, c). However, our data did not show any significant differences in average number of females, average female size, or average syncytium size in *pgip2-1* infected with BCN, but we did observe a significant reduction in the average number of males compared with the Col-0 control (Supplementary Fig. S4a–c). After RKN infection, we did not observe any changes in the average gall number or size in all tested lines (Fig. 3d–g). These combined results suggest that *PGIP1* knockout leads to hypersusceptibility of plants to CNs but not to RKNs. To confirm this differential susceptibility further, we transformed *pgip1-1* mutants with the *35S::PGIP1* overexpression construct and analysed the homozygous transgenic plants using nematode infection assays. The number of females of BCNs in transgenic plants did not differ from that of Col-0. However, one of the lines showed a significant increase in the number of males as well as the total number of nematodes (Supplementary Fig. S5a–d).

### *PGIP1* overexpression and OG treatment reduce susceptibility to cyst nematode infection but not root-knot nematode infection

**Fig. 4.**
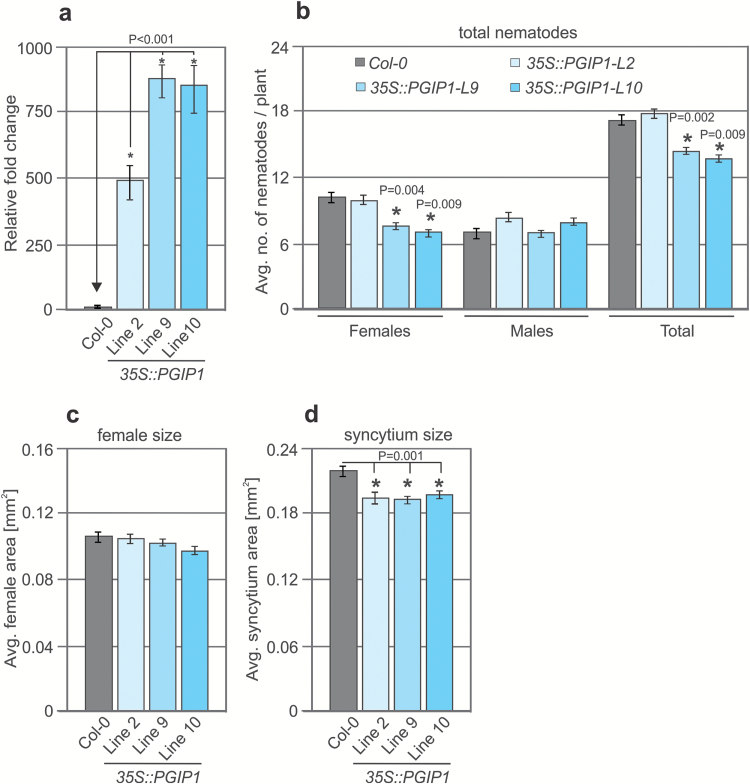
Nematode infection assays in *PGIP1* overexpression lines. (a) Three independent homozygous lines (L2, L9, and L10) overexpressing *PGIP1* (*35S::PGIP1*) were selected and analysed for changes in transcript abundance of *PGIP1*. The values represent relative fold change with the value in Col-0 plants set to 1. *UBQ5* and and *β-tubulin* were used as housekeeping genes to normalize the data. (b) Average number of females and males per plant present in Col-0 and *PGIP1* overexpression lines at 12 dpi. (c, d) Average sizes of female nematodes (c) and plant syncytia (d) in Col-0 and *PGIP1* overexpression lines at 14 dpi. (a–d) Bars represent the mean ± SE for three independent experiments. Data were analysed using Student’s *t*-test (*P*<0.05). Asterisks represent statistically significant differences from the corresponding Col-0.

As loss-of-function *PGIP1* mutants were hypersusceptible to CN infection, we hypothesized that *PGIP1* overexpression might mitigate plant susceptibility to nematode infection. We produced transgenic plants expressing *PGIP1* or *PGIP2* under the control of 35S promoters (*35S*::*PGIP1* and *35S*::*PGIP2*), performed qRT–PCR analysis of the resultant lines, and selected three homozygous lines (L2, L9, and L10) that displayed the highest *PGIP* expression levels for further experiments (Fig. 4a). We did not observe any significant phenotypic differences in the transgenic lines and the Col-0 controls. Then, 12-day-old transgenic (L2, L9, and L10) and Col-0 plants were infected with J2s of *H. schachtii*, and the results were evaluated at 14 dpi. The number of females and total number of nematodes per plant were significantly reduced for L9 and L10 compared with Col-0, but neither of these parameters differed for L2 (Fig. 4b). The average syncytium size significantly declined in all three transgenic lines compared with Col-0, but there were no significant differences in the sizes of female nematodes (Fig. 4c, d). In contrast, no significant differences were observed for any parameters in any lines overexpressing *PGIP2* (Supplementary Fig. S6a–d). Overexpression of *PGIP1* or *PGIP2* also did not affect the average gall number or size induced by RKN infection (Supplementary Fig. S7). These data suggest that overexpression of *PGIP1* leads to reduced susceptibility of plants to CNs but not to RKNs.

PGIP promotes the formation of OGs, which in turn activate host defence responses to restrict pathogen development. To evaluate whether OGs have a similar role in plant–nematode interactions, we treated the Col-0 plants with OGs and infected them with BCN. The number of females and the sizes of syncytium and females were significantly lower in plants treated with OGs than in water-treated (mock) control plants (SupplementaryFig. S8a–c), suggesting that OG-induced host defence responses are able to restrict infection of nematodes.

### *PGIP*-mediated defence responses activate indole-glucosinolate and camalexin responses

**Fig. 5.**
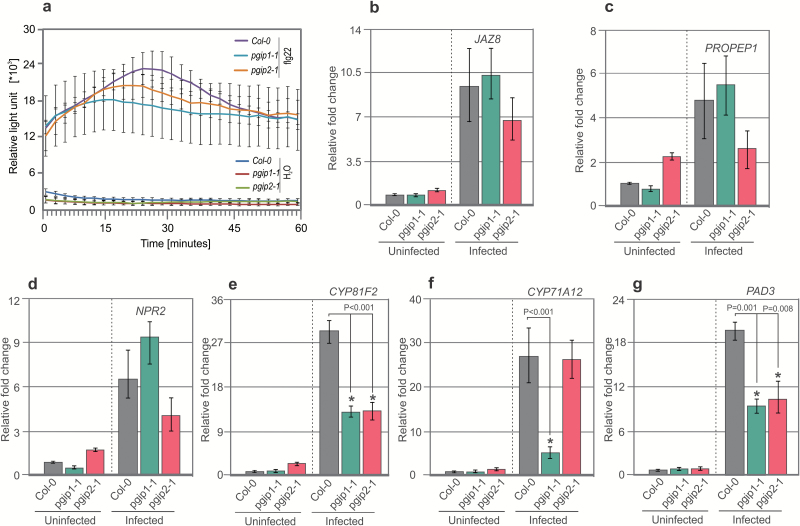
ROS production and gene expression analysis on root segments. (a) Root segments from Col-0, *pgip1-1*, and *pgip2-1* plants were treated with water or flg22, and ROS burst was measured using an L-012-based assay from 0 to 60 min. (b–g) Infected and uninfected root segments (~0.2 cm) from Col-0, *pgip1-1*, and *pgip2-1* plants were cut and gene expression was measured. For uninfected roots, data represent relative expression of the indicated genes with the value in Col-0 plants set to 1. For infected roots, data represent relative expression of the indicated genes with the value in uninfected roots set to 1. Bars represent the mean ±SE for three independent experiments. Data were analysed using Student’s *t*-test (*P*<0.05). Asterisks represent statistically significant difference from the corresponding Col-0.

Apoplastic ROS production is one of the hallmarks of pattern-triggered immunity (PTI) responses, which are activated after pathogen attack or elicitor treatment (Siddique *et al.*, 2014). To investigate whether PGIPs are involved in PTI responses and whether *pgip1* hypersusceptibility to nematode infection results from impaired ROS production, we quantitatively evaluated PTI responses by performing a luminol-based detection assay. Root segments from 2-week-old *pgip1-1* and *pgip2* mutant plants displayed the same level of ROS production in response to the immunogenic peptide flg22 as wild-type plants (Fig. 5a). These results indicate that elicitor-induced ROS production is independent of both *PGIP1* and *PGIP2*, suggesting that it plays no role in PGIP-mediated defence responses.

**Fig. 6.**
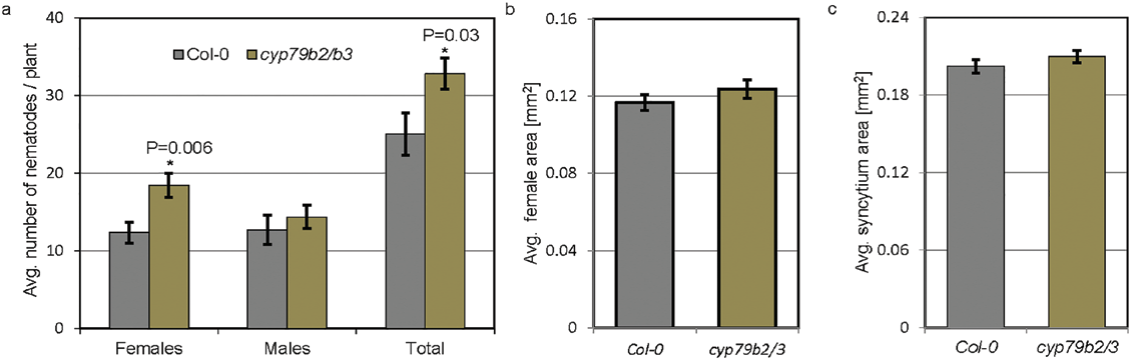
Cyst nematode infection assays in *cyp79b2/b3* lines. (a) Average number of females and males per plant present in Col-0 and *cyp79b2/b3* lines at 12 dpi. (b, c) Average sizes of female nematodes (b) and plant syncytia (c) in Col-0 and *cyp79b2/b3* lines. Data were analysed using Student’s *t*-test (*P*<0.05). Asterisks represent statistically significant differences from the corresponding Col-0.

We hypothesized that the hypersusceptibility of *pgip1* mutants might be due to impaired expression of genes in defence-related pathways. Therefore, we assessed the expression of the following genes that are strongly up-regulated during the migratory stage of infection as determined in our recent microarray data (Mendy *et al.*, 2017): *JAZ8* (Chini *et al.*, 2007), which is involved in jasmonic acid signalling; *NPR2*, a salicylic acid marker gene (Canet *et al.*, 2010); *PROPEP1*, a member of the PROPEP family that is induced in response to wounding (Huffaker *et al.*, 2006); and three genes involved in the synthesis of camalexin and indole-glucosinolate, including *CYP81F2* [encodes a cytochrome P450 involved in indol-3-yl-methyl glucosinolate catabolism (Clay *et al.*, 2009)], *CYP71B15* [PAD3, catalyses the final step in camalexin biosynthesis (Zhou *et al.*, 1999; Schuhegger *et al.*, 2006)], and *CYP71A12* [dehydrates indole-3-acetaldoxime (IAOx) to indole-3-acetonitrile (IAN) (Millet *et al.*, 2010)]. The results from qRT–PCR analyses showed no significant changes in the expression of all tested genes between *PGIP* mutants and Col-0 in uninfected roots. Next, we sampled roots at 10 hpi (migratory stage of nematode infection) and used these samples for qRT–PCR analysis. There were no changes in the expression of *JAZ8*, *PROPEP1*, or *NPR2* in *pgip1-1* or *pgip2-1* compared with Col-0 (Fig. 5b–d). In contrast, the normal up-regulation of genes involved in indole-3-glucosinolate and camalexin biosynthesis (*CYP81F2*, *CYP71A12*, and *PAD3*) was significantly impaired in *pgip1-1* compared with Col-0 (Fig. 5e–g). These results indicate that *pgip1-1* susceptibility to nematode infection results from impaired induction of camalexin and indole-3-glucosinolate biosynthesis pathways. To confirm these results, we used a double mutant *cyp79b2/b3*, which is strongly impaired in indole-glucosinolate and camalexin biosynthesis and accumulation (Zhao *et al.*, 2002; Kliebenstein *et al.*, 2005). The *cyp79b2/b3* plants were grown for 12 d *in vitro*, inoculated with cyst nematodes, and the numbers of males and females were counted. The number of females increased significantly in *cyp79b2/b3* compared with Col-0 (Fig. 6a). However, we did not observe any significant differences in the average sizes of females and syncytia in *cyp79b2/b3* and Col-0 (Fig. 6b, c). Taken together, these results suggested that BCN migration within roots induced camalexin and indole-glucosinolate biosynthesis pathways in a PGIP1-dependent manner, which restricted the number of nematodes.

## Discussion

In the present study, we established a molecular framework for PGIP regulation and downstream signalling in Arabidopsis during CN and RKN parasitism. We first analysed the expression of *PGIP1* and *PGIP2* in response to BCN and RKN infection and found commonalities, but also differences between two nematode species. We found that expression of both *PGIP1* and *PGIP2* is induced during migratory stages of BCN infection. This expression was localized to the infection zone close to the head of nematodes, suggesting that the induction is highly specific to infection. In contrast to BCN, RKN migration inside the roots did not induce *PGIP* expression at 1 dpi (migratory stage), unravelling what may be a key difference in *PGIP* regulation between the two nematode species. Previously, Ferrari *et al.* 2003 showed that expression of *PGIP1* and *PGIP2* is induced by wounding in leaves and we also observed a highly specific activation of *PGIP* gene expression in roots upon wounding. Therefore, the difference in *PGIP* expression during migratory stages is likely to be due to a difference in the migration style of CNs versus RKNs. Whereas RKNs migrate intercellularly and cause little damage, CNs migrate intracellularly and cause severe damage to root cells (Wyss and Zunke, 1986; Wyss *et al.*, 1992). The hypothesis that RKN do not cause damage during their migration inside the root is also in line with a recent study showing that Arabidopsis lines with altered damage perception do not show any change in susceptibility to RKN (Teixeira *et al.*, 2016).

The RKN *M. incognita* encodes a PG (MI-PG-1) that is secreted into the host tissue to weaken the plant cell wall during nematode penetration and intercellular migration (Jaubert *et al.*, 2002). However, our experiments to identify a functional PG in CN have proven unsuccessful. These observations raise the question of whether PG secretion by nematodes (if any) has a role in activation of *PGIP* expression during nematode infection of plant roots. We did not observe any *PGIP* expression during the migratory stage of RKN infection and CNs do not appear to encode a PG. Therefore, we postulate that *PGIP* induction during nematode infection is independent of nematode-derived PGs, at least during the migration stage. This hypothesis is consistent with observations that MI-PG-1 is an exo-PG, which are not usually inhibited by PGIPs (Jaubert *et al.*, 2002; Schacht *et al.*, 2011).

OG-mediated resistance to the necrotrophic fungal pathogen *Botrytis cinerea* is independent of salicylic acid, jasmonic acid, and ethylene, but requires PAD3, which catalyses the final step in camalexin biosynthesis (Ferrari *et al.*, 2007). Here, we found that knocking out or overexpressing *PGIP1* significantly increased or decreased, respectively, the susceptibility of plants to CN. Further, pre-treatment of plants with OGs led to a significant reduction in nematode infection. Based on these data, we propose that activation of *PGIP* in response to CN infection promotes the formation of active OG elicitor, which in turn activates the expression of genes involved in indole-glucosinolate and camalexin biosynthesis. Indeed, we found that up-regulation of three key indole-glucosinolate and camalexin biosynthesis genes (*CYP71A12*, *CYP71B15/PAD3*, and *CYP81F2*) in response to BCN infection was impaired in *pgip* mutants (especially in *pgip1*) compared with Col-0 control plants. Conversely, the double mutant *cyp79b2/b3*, which is deficient in camalexin and indole-glucosinolate production, displays enhanced susceptibility to CN (Zhao *et al.*, 2002; Kliebenstein *et al.*, 2005). The relevance of camalexin in CN infection is further evidenced by the fact that loss-of-function *pad3* mutants are more susceptible to the BCN (Ali *et al.*, 2013). The impaired up-regulation of camalexin and indole-glucosinolate genes is only partial in *pgip* mutants, which is probably due to the functional redundancy in this gene family. It is also plausible that these genes are regulated in both a PGIP-dependent and a PGIP-independent manner during CN parasitism. RKN invasion of the Arabidopsis root has been shown to induce *PAD3* expression during migratory stages of infection. In addition, mutants that are impaired in indole-glucosinolate or camalexin biosynthesis are hypersusceptible to RKN (Teixeira *et al.*, 2016). These previous observations, together with the fact that we did not observe any *PGIP* expression during early stages of infection, suggest that camalexin and indole-glucosinolate biosynthesis is regulated in a PGIP-independent manner during plant–RKN interactions.

The consistent expression of *PGIP* genes in syncytia and giant cells during biotrophic stages of parasitism suggests that these genes may have a role in nematode parasitism other than activation of PTI-like defence responses. PGIPs have been shown to interact with partially or completely de-esterified homogalacturonan (HG) in pectin, and protect it from the hydrolysing activity of plant or pathogen PGs (Spadoni *et al.*, 2006). Thus, the PGIP expression level probably reflects a contribution to the mechanical properties of the cell wall related to growth and development. Previous studies showed that HG in the cell walls of younger syncytia (5 dpi) is highly de-esterified compared with that of older syncytia (15 dpi). In contrast, highly methylesterified HG was abundant in the cell wall of younger (7 dpi) and older (14 dpi) giant cells (Davies *et al.*, 2012; Wieczorek *et al.*, 2014). Although the syncytium and giant cells perform the same function, they have different ontogenies, which might underlie the differences in methylesterification of younger feeding sites associated with CNs or RKNs.

The syncytium expands through dissolution of the cell wall and fusion of root cells. During cell wall expansion, the wall is locally degraded and modified, which ultimately leads to local wall strengthening and thickening (Siddique *et al.*, 2012; Wieczorek *et al.*, 2014). In contrast, giant cells grow via repeated nuclear division without cytokinesis. Therefore, extensive de-esterification of the cell wall at 5 dpi may facilitate wall degradation and promote syncytium expansion. Conversely, a higher level of methylesterification in older feeding sites of both CNs and RKNs may provide higher strength and flexibility to the cell wall, which may contribute to the capacity of these feeding sites to sustain high turgor pressure during parasitism (Böckenhoff and Grundler, 1994). Based on these observations, we hypothesize that the high *PGIP* expression in younger syncytia at 5 dpi plays a role in regulation of local cell wall degradation by allowing PGIPs to bind directly PGs (of plant or nematode origin) and HG, protecting the cell wall from further degradation. This hypothesis is consistent with our observations that *PGIP1* knockout or overexpression significantly increases or reduces, respectively, the average size of the syncytium. Cell wall degradation slows down as the syncytium expands and reaches its maximum size, which was accompanied by a reduction in *PGIP* expression levels. In contrast, *PGIP1* was consistently and highly expressed in galls/giant cells throughout the sedentary stages of nematode development, which may protect the cell walls from enzymatic degradation by blocking de-esterified HG. However, no significant phenotypic differences were observed for RKN infection in any of the lines we tested, possibly due to functional redundancy within the *PGIP* gene family.

In conclusion, this study identified the molecular mechanism underlying PGIP-mediated damage-associated responses during CN and RKN parasitism of plants. We showed that differential regulation of *PGIP* genes occurs during CN and RKN invasion of roots, probably associated with differences in nematode migration and feeding habits. We also determined that PGIP regulates camalexin and indole-glucosinolate biosynthetic pathways in an infection-specific manner. These results provide new insights into the functional mechanisms underlying nematode parasitism. Clarifying further details of damage-associated pathways in plant–nematode interactions may lead to novel control measures for this important plant parasite.

## Supplementary data

Supplementary data are available at *JXB* online.

Fig. S1. Genotyping of SALK T-DNA insertion line.

Fig. S2. RT–PCR analysis of gene expression profiles in Col-0 and knockout mutants.

Fig. S3. Cyst nematode infection assays in *PGIP1* (*pgip1-2*) mutant lines.

Fig. S4. Cyst nematode infection assays in *PGIP2-1* mutant lines.

Fig. S5. Cyst nematode infection assays in complementation lines for *PGIP1* (*35S::PGIP1/pgip1-1*) mutants.

Fig. S6. Cyst nematode infection assays in *PGIP2* overexpression lines.

Fig. S7. Root-knot nematode infection assays in *PGIP1* and *PGIP2* overexpression lines.

Fig. S8. Cyst nematode infection assays upon OG treatment.

Table S1. Primer sequences used in this work.

Table S2. Overview of *PGIP1* and *PGIP2* expression patterns in published transcriptomic data.

## Supplementary Material

Supplementary Figures and Tables

## References

[CIT0001] Ali MA, Abbas A, Kreil DP, Bohlmann H 2013 Overexpression of the transcription factor RAP2.6 leads to enhanced callose deposition in syncytia and enhanced resistance against the beet cyst nematode *Heterodera schachtii* in Arabidopsis roots. BMC Plant Biology 13, 47. 23510309 10.1186/1471-2229-13-47 PMC3623832

[CIT0002] Barcala M, García A, Cabrera J, Casson S, Lindsey K, Favery B, García-Casado G, Solano R, Fenoll C, Escobar C 2010 Early transcriptomic events in microdissected Arabidopsis nematode-induced giant cells. The Plant Journal 61, 698–712. 20003167 10.1111/j.1365-313X.2009.04098.x

[CIT0003] Bellincampi D, Dipierro N, Salvi G, Cervone F, De Lorenzo G 2000 Extracellular H(2)O(2) induced by oligogalacturonides is not involved in the inhibition of the auxin-regulated rolB gene expression in tobacco leaf explants. Plant Physiology 122, 1379–1385. 10759534 10.1104/pp.122.4.1379 PMC58973

[CIT0004] Benedetti M, Pontiggia D, Raggi S 2015 Plant immunity triggered by engineered *in vivo* release of oligogalacturonides, damage-associated molecular patterns. Proceedings of the National Academy of Sciences, USA 112, 5533–5538. 10.1073/pnas.1504154112 PMC4418913 25870275

[CIT0005] Bishop PD, Makus DJ, Pearce G, Ryan CA 1981 Proteinase inhibitor-inducing factor activity in tomato leaves resides in oligosaccharides enzymically released from cell walls. Proceedings of the National Academy of Sciences, USA 78, 3536–3540. 10.1073/pnas.78.6.3536 PMC319604 16593033

[CIT0006] Böckenhoff A, Grundler FMW 1994 Studies on the nutrient uptake by the beet cyst nematode *Heterodera schachtii* by in situ microinjection of fluorescent probes into the feeding structures in *Arabidopsis thaliana*. Parasitology 109, 249–255.

[CIT0007] Cabanne C, Donèche B 2002 Purification and characterization of two isozymes of polygalacturonase from *Botrytis cinerea*. Effect of calcium ions on polygalacturonase activity. Microbiological Research 157, 183–189. 12398287 10.1078/0944-5013-00147

[CIT0008] Cabrera J, Bustos R, Favery B, Fenoll C, Escobar C 2014 NEMATIC: a simple and versatile tool for the insilico analysis of plant–nematode interactions. Molecular Plant Pathology 15, 627–636. 24330140 10.1111/mpp.12114 PMC6638708

[CIT0009] Canet JV, Dobón A, Roig A, Tornero P 2010 Structure–function analysis of npr1 alleles in Arabidopsis reveals a role for its paralogs in the perception of salicylic acid. Plant, Cell and Environment 33, 1911–1922. 10.1111/j.1365-3040.2010.02194.x 20561252

[CIT0010] Celorio-Mancera MD, Allen ML, Powell AL 2008 Polygalacturonase causes lygus-like damage on plants: cloning and identification of western tarnished plant bug (*Lygus hesperus*) polygalacturonases secreted during feeding. Arthropod-Plant Interactions 2, 215–225.

[CIT0011] Celorio-Mancera Mde L, Carl Greve L, Teuber LR, Labavitch JM 2009 Identification of endo- and exo-polygalacturonase activity in *Lygus hesperus* (Knight) salivary glands. Archives of Insect Biochemistry and Physiology 70, 122–135. 19085947 10.1002/arch.20282

[CIT0012] Chini A, Fonseca S, Fernández G 2007 The JAZ family of repressors is the missing link in jasmonate signalling. Nature 448, 666–671. 17637675 10.1038/nature06006

[CIT0013] Clay NK, Adio AM, Denoux C, Jander G, Ausubel FM 2009 Glucosinolate metabolites required for an Arabidopsis innate immune response. Science 323, 95–101. 19095898 10.1126/science.1164627 PMC2630859

[CIT0014] Clough SJ, Bent AF 1998 Floral dip: a simplified method for *Agrobacterium*-mediated transformation of *Arabidopsis thaliana*. The Plant Journal 16, 735–743. 10069079 10.1046/j.1365-313x.1998.00343.x

[CIT0015] Côté F, Hahn MG 1994 Oligosaccharins: structures and signal transduction. Plant Molecular Biology 26, 1379–1411. 7858196 10.1007/BF00016481

[CIT0016] Curtis MD, Grossniklaus U 2003 A gateway cloning vector set for high-throughput functional analysis of genes in planta. Plant Physiology 133, 462–469. 14555774 10.1104/pp.103.027979 PMC523872

[CIT0017] Davies LJ, Lilley CJ, Paul Knox J, Urwin PE 2012 Syncytia formed by adult female *Heterodera schachtii* in *Arabidopsis thaliana* roots have a distinct cell wall molecular architecture. New Phytologist 196, 238–246. 22803660 10.1111/j.1469-8137.2012.04238.x

[CIT0018] Davis KR, Darvill AG, Albersheim P, Dell A 1986 Host–pathogen interactions: XXIX. Oligogalacturonides released from sodium polypectate by endopolygalacturonic acid lyase are elicitors of phytoalexins in soybean. Plant Physiology 80, 568–577. 16664663 10.1104/pp.80.2.568 PMC1075156

[CIT0019] de Boer JM, Davis EL, Hussey RS, Popeijus H, Smant G, Baum TJ 2002 Cloning of a putative pectate lyase gene expressed in the subventral esophageal glands of *Heterodera glycines*. Journal of Nematology 34, 9–11. 19265900 PMC2620537

[CIT0020] de Boer JM, Yan Y, Wang X, Smant G, Hussey RS, Davis EL, Baum TJ 1999 Developmental expression of secretory beta-1,4-endoglucanases in the subventral esophageal glands of *Heterodera glycines*. Molecular Plant-Microbe Interactions 12, 663–669. 10432634 10.1094/MPMI.1999.12.8.663

[CIT0021] Decraemer W, Hunt DJ 2006 Structure and classification. In: Perry RN, Moens M, eds. Plant nematology, Wallingford, UK: CABI, 187–209.

[CIT0022] Di Matteo A, Federici L, Mattei B 2003 The crystal structure of polygalacturonase-inhibiting protein (PGIP), a leucine-rich repeat protein involved in plant defense. Proceedings of the National Academy of Sciences, USA 100, 10124–10128. 10.1073/pnas.1733690100 PMC187787 12904578

[CIT0023] Favaron F, Alghisi P, Marciano P 1992 Characterization of 2 *Sclerotinia sclerotiorum* polygalacturonases with different abilities to elicit glyceollin in soybean. Plant Science 83, 7–13.

[CIT0024] Ferrari S, Galletti R, Denoux C, De Lorenzo G, Ausubel FM, Dewdney J 2007 Resistance to *Botrytis cinerea* induced in Arabidopsis by elicitors is independent of salicylic acid, ethylene, or jasmonate signaling but requires PHYTOALEXIN DEFICIENT3. Plant Physiology 144, 367–379. 17384165 10.1104/pp.107.095596 PMC1913806

[CIT0025] Ferrari S, Vairo D, Ausubel FM, Cervone F, De Lorenzo G 2003 Tandemly duplicated Arabidopsis genes that encode polygalacturonase-inhibiting proteins are regulated coordinately by different signal transduction pathways in response to fungal infection. The Plant Cell 15, 93–106. 12509524 10.1105/tpc.005165 PMC143454

[CIT0026] Fosu-Nyarko J, Nicol P, Naz F, Gill R, Jones MG 2016 Analysis of the transcriptome of the infective stage of the beet cyst nematode, *H. schachtii*. PLoS One 11, e0147511. 26824923 10.1371/journal.pone.0147511 PMC4733053

[CIT0027] Galletti R, Denoux C, Gambetta S, Dewdney J, Ausubel FM, De Lorenzo G, Ferrari S 2008 The AtrbohD-mediated oxidative burst elicited by oligogalacturonides in Arabidopsis is dispensable for the activation of defense responses effective against *Botrytis cinerea*. Plant Physiology 148, 1695–1706. 18790995 10.1104/pp.108.127845 PMC2577270

[CIT0028] Hahn MG, Darvill AG, Albersheim P 1981 Host–pathogen interactions: XIX. The endogenous elicitor, a fragment of a plant cell wall polysaccharide that elicits phytoalexin accumulation in soybeans. Plant Physiology 68, 1161–1169. 16662068 10.1104/pp.68.5.1161 PMC426062

[CIT0029] Holbein J, Grundler FM, Siddique S 2016 Plant basal resistance to nematodes: an update. Journal of Experimental Botany 67, 2049–2061. 26842982 10.1093/jxb/erw005

[CIT0030] Hofmann J, Grundler FMW 2007 Identification of reference genes for qRT-PCR studies of gene expression in giant cells and syncytia induced in *Arabidopsis thaliana* by *Meloidogyne incognita* and *Heterodera schachtii*. Nematology 9, 317–323.

[CIT0031] Huang Q, Allen C 2000 Polygalacturonases are required for rapid colonization and full virulence of *Ralstonia solanacearum* on tomato plants. Physiological and Molecular Plant Pathology 57, 77–83.

[CIT0032] Huffaker A, Pearce G, Ryan CA 2006 An endogenous peptide signal in Arabidopsis activates components of the innate immune response. Proceedings of the National Academy of Sciences, USA 103, 10098–10103. 10.1073/pnas.0603727103 PMC1502512 16785434

[CIT0033] Jammes F, Lecomte P, de Almeida-Engler J, Bitton F, Martin-Magniette ML, Renou JP, Abad P, Favery B 2005 Genome-wide expression profiling of the host response to root-knot nematode infection in Arabidopsis. The Plant Journal 44, 447–458. 16236154 10.1111/j.1365-313X.2005.02532.x

[CIT0034] Jaubert S, Laffaire JB, Abad P, Rosso MN 2002 A polygalacturonase of animal origin isolated from the root-knot nematode *Meloidogyne incognita*. FEBS Letters 522, 109–112. 12095628 10.1016/s0014-5793(02)02906-x

[CIT0035] Kalunke RM, Tundo S, Benedetti M, Cervone F, De Lorenzo G, D’Ovidio R 2015 An update on polygalacturonase-inhibiting protein (PGIP), a leucine-rich repeat protein that protects crop plants against pathogens. Frontiers in Plant Science 6, 146. 25852708 10.3389/fpls.2015.00146 PMC4367531

[CIT0036] Kester HC, Visser J 1990 Purification and characterization of polygalacturonases produced by the hyphal fungus *Aspergillus niger*. Biotechnology and Applied Biochemistry 12, 150–160. 2331322

[CIT0037] Kliebenstein DJ, Rowe HC, Denby KJ 2005 Secondary metabolites influence Arabidopsis/Botrytis interactions: variation in host production and pathogen sensitivity. The Plant Journal 44, 25–36. 16167893 10.1111/j.1365-313X.2005.02508.x

[CIT0038] Kyndt T, Vieira P, Gheysen G, de Almeida-Engler J 2013 Nematode feeding sites: unique organs in plant roots. Planta 238, 807–818. 23824525 10.1007/s00425-013-1923-z

[CIT0039] Laurema S, Varis AL, Miettinen H 1985 Studies on enzymes in the salivary glands of *Lygus rugulipennis* (Hemiptera, Miridae). Insect Biochemistry 15, 211–224.

[CIT0040] Livak KJ, Schmittgen TD 2001 Analysis of relative gene expression data using real-time quantitative PCR and the 2(-Delta Delta C(T)) method. Methods 25, 402–408. 11846609 10.1006/meth.2001.1262

[CIT0041] Maldonado MC, Strasser de Saad AM 1998 Production of pectinesterase and polygalacturonase by *Aspergillus niger* in submerged and solid state systems. Journal of Industrial Microbiology and Biotechnology 20, 34–38. 9523455 10.1038/sj.jim.2900470

[CIT0042] Mendy B, Wang’ombe MW, Radakovic ZS, Holbein J, Ilyas M, Chopra D, Holton N, Zipfel C, Grundler FM, Siddique S 2017 Arabidopsis leucine-rich repeat receptor-like kinase NILR1 is required for induction of innate immunity to parasitic nematodes. PLoS Pathogens 13, e1006284. 28406987 10.1371/journal.ppat.1006284 PMC5391088

[CIT0043] Millet YA, Danna CH, Clay NK, Songnuan W, Simon MD, Werck-Reichhart D, Ausubel FM 2010 Innate immune responses activated in Arabidopsis roots by microbe-associated molecular patterns. The Plant Cell 22, 973–990. 20348432 10.1105/tpc.109.069658 PMC2861455

[CIT0044] Nagel CW, Vaughn RH 1961 The characteristics of a polygalacturonase produced by *Bacillus polymyxa*. Archives of Biochemistry and Biophysics 93, 344–352. 13727437 10.1016/0003-9861(61)90277-6

[CIT0045] Nicol JM, Turner SJ, Coyne DL 2011 Current nematode threats to world agriculture. In: Jones J, Gheysen G, Fenoll C, eds. Genomics and molecular genetics of plant–nematode interactions. Dordrecht: Springer Netherlands, 21–43.

[CIT0046] Nothnagel EA, McNeil M, Albersheim P, Dell A 1983 Host–pathogen interactions: XXII. A galacturonic acid oligosaccharide from plant cell walls elicits phytoalexins. Plant Physiology 71, 916–926. 16662929 10.1104/pp.71.4.916 PMC1066144

[CIT0047] Oeser B, Heidrich PM, Müller U, Tudzynski P, Tenberge KB 2002 Polygalacturonase is a pathogenicity factor in the *Claviceps purpurealrye* interaction. Fungal Genetics and Biology 36, 176–186. 12135573 10.1016/s1087-1845(02)00020-8

[CIT0048] Pfaffl MW 2001 A new mathematical model for relative quantification in real-time RT-PCR. Nucleic Acids Research 29, e45. 11328886 10.1093/nar/29.9.e45 PMC55695

[CIT0049] Rahman MB, Joslyn MA 1953a The hydrolysis of pectic acid by purified fungal polygalacturonase. Journal of Food Science 18, 308–318.

[CIT0050] Rahman MB, Joslyn MA 1953b Properties of purified fungal polygalacturonase. Journal of Food Science 18, 301–304.

[CIT0051] Rasul S, Dubreuil-Maurizi C, Lamotte O, Koen E, Poinssot B, Alcaraz G, Wendehenne D, Jeandroz S 2012 Nitric oxide production mediates oligogalacturonide-triggered immunity and resistance to *Botrytis cinerea* in *Arabidopsis thaliana*. Plant, Cell and Environment 35, 1483–1499. 10.1111/j.1365-3040.2012.02505.x 22394204

[CIT0052] Reymond-Cotton P, Fraissinet-Tachet L, Fèvre M 1996 Expression of the *Sclerotinia sclerotiorum* polygalacturonase pg1 gene: possible involvement of CREA in glucose catabolite repression. Current Genetics 30, 240–245. 8753653 10.1007/s002940050127

[CIT0053] Rodriguez-Palenzuela P, Burr TJ, Collmer A 1991 Polygalacturonase is a virulence factor in *Agrobacterium tumefaciens* biovar 3. Journal of Bacteriology 173, 6547–6552. 1655716 10.1128/jb.173.20.6547-6552.1991 PMC208991

[CIT0054] Schacht T, Unger C, Pich A, Wydra K 2011 Endo- and exopolygalacturonases of *Ralstonia solanacearum* are inhibited by polygalacturonase-inhibiting protein (PGIP) activity in tomato stem extracts. Plant Physiology and Biochemistry 49, 377–387. 21367611 10.1016/j.plaphy.2011.02.001

[CIT0055] Schuhegger R, Nafisi M, Mansourova M, Petersen BL, Olsen CE, Svatos A, Halkier BA, Glawischnig E 2006 CYP71B15 (PAD3) catalyzes the final step in camalexin biosynthesis. Plant Physiology 141, 1248–1254. 16766671 10.1104/pp.106.082024 PMC1533948

[CIT0056] Siddique S, Grundler FMW 2015 Metabolism in nematode feeding sites. Advances in Botanical Research 73, 119–138.

[CIT0057] Siddique S, Matera C, Radakovic ZS, Hasan MS, Gutbrod P, Rozanska E, Sobczak M, Torres MA, Grundler FM 2014 Parasitic worms stimulate host NADPH oxidases to produce reactive oxygen species that limit plant cell death and promote infection. Science Signaling 7, ra33. 24714570 10.1126/scisignal.2004777

[CIT0058] Siddique S, Radakovic ZS, De La Torre CM 2015 A parasitic nematode releases cytokinin that controls cell division and orchestrates feeding site formation in host plants. Proceedings of the National Academy of Sciences, USA 112, 12669–12674. 10.1073/pnas.1503657112 PMC4611629 26417108

[CIT0059] Siddique S, Sobczak M, Tenhaken R, Grundler FM, Bohlmann H 2012 Cell wall ingrowths in nematode induced syncytia require UGD2 and UGD3. PLoS One 7, e41515. 22848518 10.1371/journal.pone.0041515 PMC3406070

[CIT0060] Smant G, Stokkermans JPWG, Yan YT 1998 Endogenous cellulases in animals: isolation of beta-1,4-endoglucanase genes from two species of plant-parasitic cyst nematodes. Proceedings of the National Academy of Sciences, USA 95, 4906–4911. 10.1073/pnas.95.9.4906 PMC20186 9560201

[CIT0061] Spadoni S, Zabotina O, Di Matteo A, Mikkelsen JD, Cervone F, De Lorenzo G, Mattei B, Bellincampi D 2006 Polygalacturonase-inhibiting protein interacts with pectin through a binding site formed by four clustered residues of arginine and lysine. Plant Physiology 141, 557–564. 16648220 10.1104/pp.106.076950 PMC1475430

[CIT0062] Strong FE, Kruitwagen EC 1968 Polygalacturonase in salivary apparatus of *Lygus hesperus* (Hemiptera). Journal of Insect Physiology 14, 1113–1119.

[CIT0063] Szakasits D, Heinen P, Wieczorek K, Hofmann J, Wagner F, Kreil DP, Sykacek P, Grundler FM, Bohlmann H 2009 The transcriptome of syncytia induced by the cyst nematode *Heterodera schachtii* in Arabidopsis roots. The Plant Journal 57, 771–784. 18980640 10.1111/j.1365-313X.2008.03727.x PMC2667683

[CIT0064] Teixeira MA, Wei L, Kaloshian I 2016 Root-knot nematodes induce pattern-triggered immunity in *Arabidopsis thaliana* roots. New Phytologist 211, 276–287. 26892116 10.1111/nph.13893

[CIT0065] Themmen AP, Tucker GA, Grierson D 1982 Degradation of isolated tomato cell walls by purified polygalacturonase in vitro. Plant Physiology 69, 122–124. 16662142 10.1104/pp.69.1.122 PMC426158

[CIT0066] Vanholme B, van Thuyne W, Vanhouteghem K, de Meutter J, Cannoot B, Gheysen G 2007 Molecular characterization and functional importance of pectate lyase secreted by the cyst nematode *Heterodera schachtii*. Molecular Plant Pathology 8, 267–278. 20507498 10.1111/j.1364-3703.2007.00392.x

[CIT0067] Veronico P, Melillo MT, Saponaro C, Leonetti P, Picardi E, Jones JT 2011 A polygalacturonase-inhibiting protein with a role in pea defence against the cyst nematode *Heterodera goettingiana*. Molecular Plant Pathology 12, 275–287. 21355999 10.1111/j.1364-3703.2010.00671.x PMC6640500

[CIT0068] Whitehead MP, Shieh MT, Cleveland TE, Cary JW, Dean RA 1995 Isolation and characterization of polygalacturonase genes (pecA and pecB) from *Aspergillus flavus*. Applied and Environmental Microbiology 61, 3316–3322. 7574642 10.1128/aem.61.9.3316-3322.1995 PMC167612

[CIT0069] Wieczorek K, Elashry A, Quentin M, Grundler FM, Favery B, Seifert GJ, Bohlmann H 2014 A distinct role of pectate lyases in the formation of feeding structures induced by cyst and root-knot nematodes. Molecular Plant-Microbe Interactions 27, 901–912. 24905398 10.1094/MPMI-01-14-0005-R

[CIT0070] Wyss U, Grundler FMW, Munch A 1992 The parasitic behaviour of second-stage juveniles of *Meloidogyne incognita* in roots of *Arabidopsis thaliana*. Nematologica 38, 98–111.

[CIT0071] Wyss U, Zunke U 1986 Observations on the behavior of second stage juveniles of *Heterodera schachtii* inside host roots. Revue de Nématologie 9, 153–165.

[CIT0072] Yamaguchi YL, Suzuki R, Cabrera J 2017 Root-knot and cyst nematodes activate procambium-associated genes in Arabidopsis roots. Frontiers in Plant Science 8, 1195. 28747918 10.3389/fpls.2017.01195 PMC5506325

[CIT0073] Zhao Y, Hull AK, Gupta NR, Goss KA, Alonso J, Ecker JR, Normanly J, Chory J, Celenza JL 2002 Trp-dependent auxin biosynthesis in Arabidopsis: involvement of cytochrome P450s *CYP79B2* and *CYP79B3*. Genes and Development 16, 3100–3112. 12464638 10.1101/gad.1035402 PMC187496

[CIT0074] Zhou N, Tootle TL, Glazebrook J 1999 Arabidopsis PAD3, a gene required for camalexin biosynthesis, encodes a putative cytochrome P450 monooxygenase. The Plant Cell 11, 2419–2428. 10590168 10.1105/tpc.11.12.2419 PMC144139

