# Supplementary material for: Damage-associated responses of the host contribute to defence against cyst nematodes but not root-knot nematodes
Source: J Exp Bot. 2017 Oct 19;68(21-22):5949–60. doi: 10.1093/jxb/erx374 (PMC5854129; doi:10.1093/jxb/erx374)
Supplement: Supplementary Figures and Tables [file erx374_suppl_supplementary_figures_and_tables.pdf]

Fig. S1: Genotyping for *pgip1-1* mutants. Genomic DNA of Col-0 or knockout lines (*pgip1-1;* SALK_001662.33.10.x*)* was PCR amplified using Left primer (LP), Right Primer (RP), and border primer (BP or LB1.3). The presence or absence of intact wild-type allele is shown. Expected size for wild-type (LP+RP) was 1117bp and for *pgip1-1* (BP+RP) 511-811bp. Primer sequences are given in Table S1.

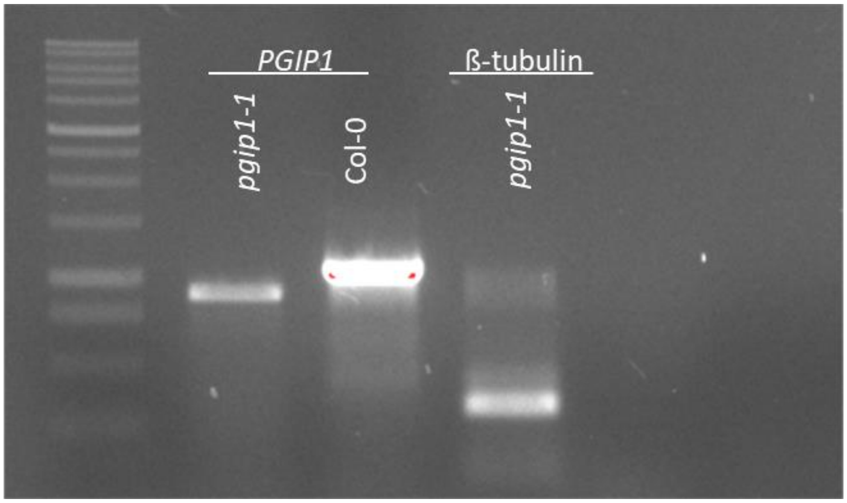

Fig. S2: **RT-PCR for presence or absence of gene expression in Col-0 or knockout mutants.** RNA from Col-0 or knockout lines (*pgip1-1, pgip1-2, pgip2-1*) was extracted to synthesize single stranded cDNA. The presence or absence of expression is shown using primers given in Table S2. UBQ5 was used as a positive control.

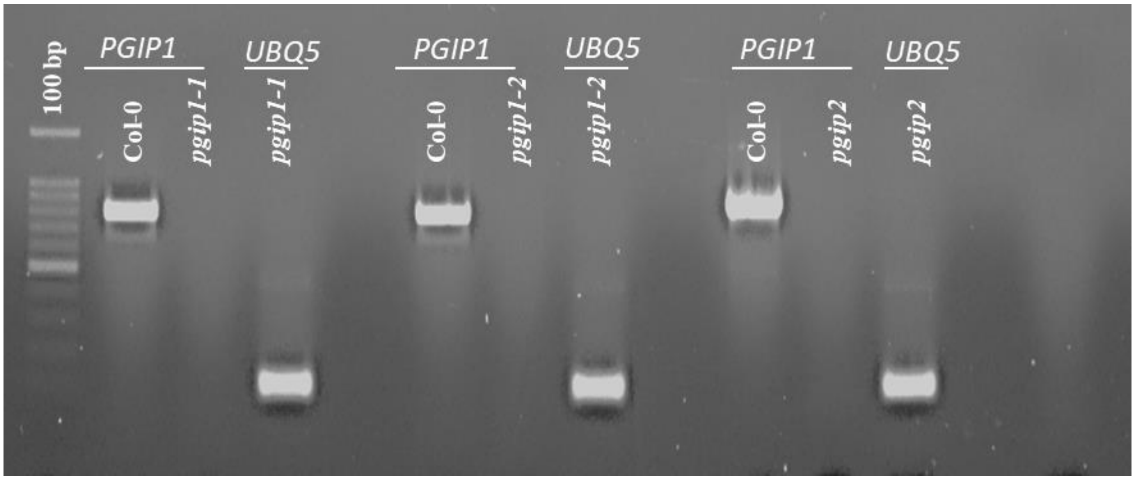

Fig. S3: **Cyst nematode infection assays in PGIP1 (*pgip1-2*) mutant lines**. (a) Average number of females and males per plant present in Col-0 and *pgip1-2* mutant lines at 12 dai. (b, c) Average sizes of female nematodes (b) and plant syncytia (c) in Col-0 and *pgip1-2* mutant lines. Bars represent mean ± SE for three independent experiments. Data were analysed using student's T-test ($P < 0.05$). Asterisks represent statistically significant difference to corresponding Col-0.

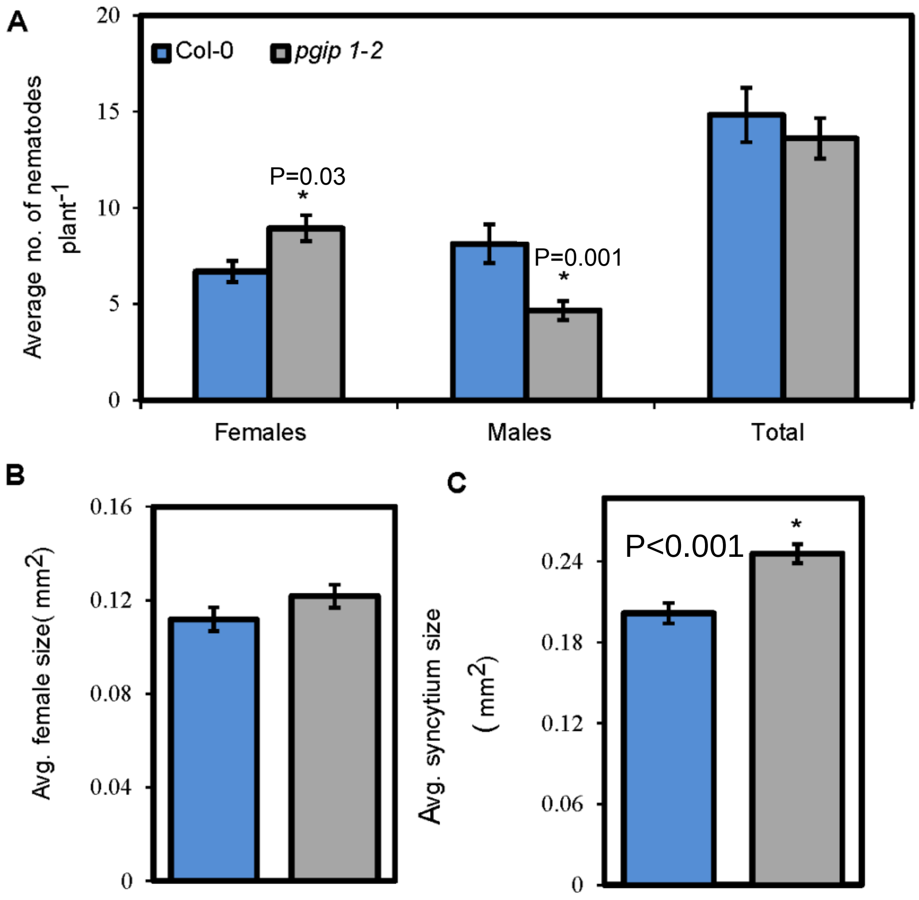

Fig. S4: Cyst nematode infection assays in PGIP2 (*pgip2-1*) mutant lines. (a) Average number of females and males per plant present in Col-0 and *pgip2-1* mutant lines at 12 dai. (b, c) Average sizes of female nematodes (b) and plant syncytia (c) in Col-0 and *pgip1-1* mutant lines. Bars represent mean ± SE for three independent experiments. Data were analysed using student's T-test ($P < 0.05$). Asterisks represent statistically significant difference to corresponding Col-0.

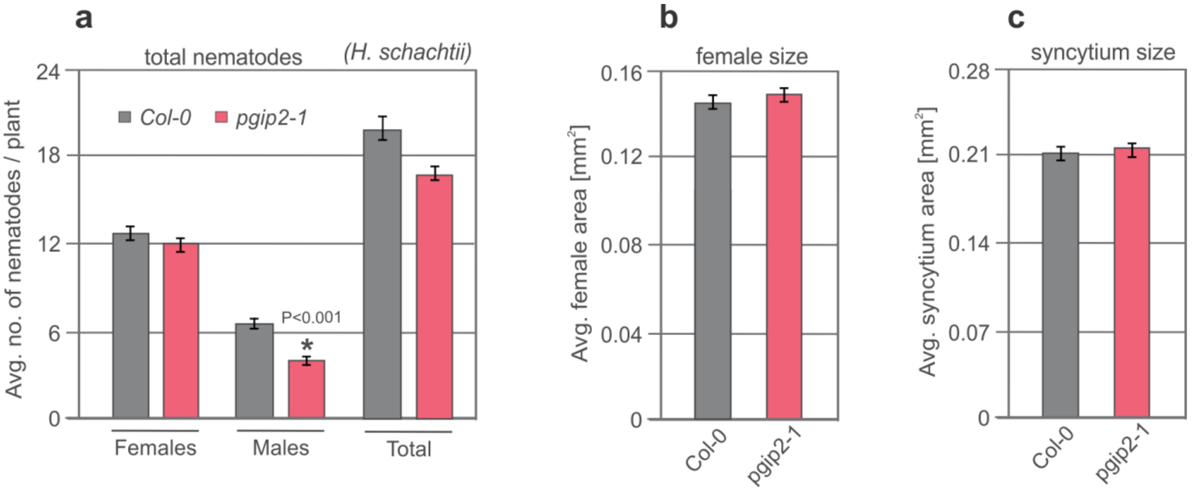

Fig. S5: Cyst nematode infection assays in complementation lines for *PGIP1* (*35S::PGIP1/pgip1-1*) mutant lines. Two independent homozygous lines overexpressing *PGIP1* in *pgip1-1* background (*35S::PGIP1/pgip1-1-L1*, *35S::PGIP1/pgip1-1-L2*) were selected and analysed for changes in transcript abundance of PGIP. *UBQ5* and *β-tubulin* were used as housekeeping genes to normalize the data. The values represent relative fold change in roots of transgenic lines with the value in uninfected Col-0 root set to one. (b, d) Average area of female nematodes (b) and plant syncytia (d) in Col-0 and PGIP1 overexpression lines at 14 dai. (c) Average number of females and males per plant present in Col-0 and PGIP1 overexpression lines at 12 dai. (b-d) Bars represent mean ± SE for three independent experiments. Data were analysed using student's T-test ($p < 0.05$). Asterisks represent statistically significant difference to corresponding Col-0.

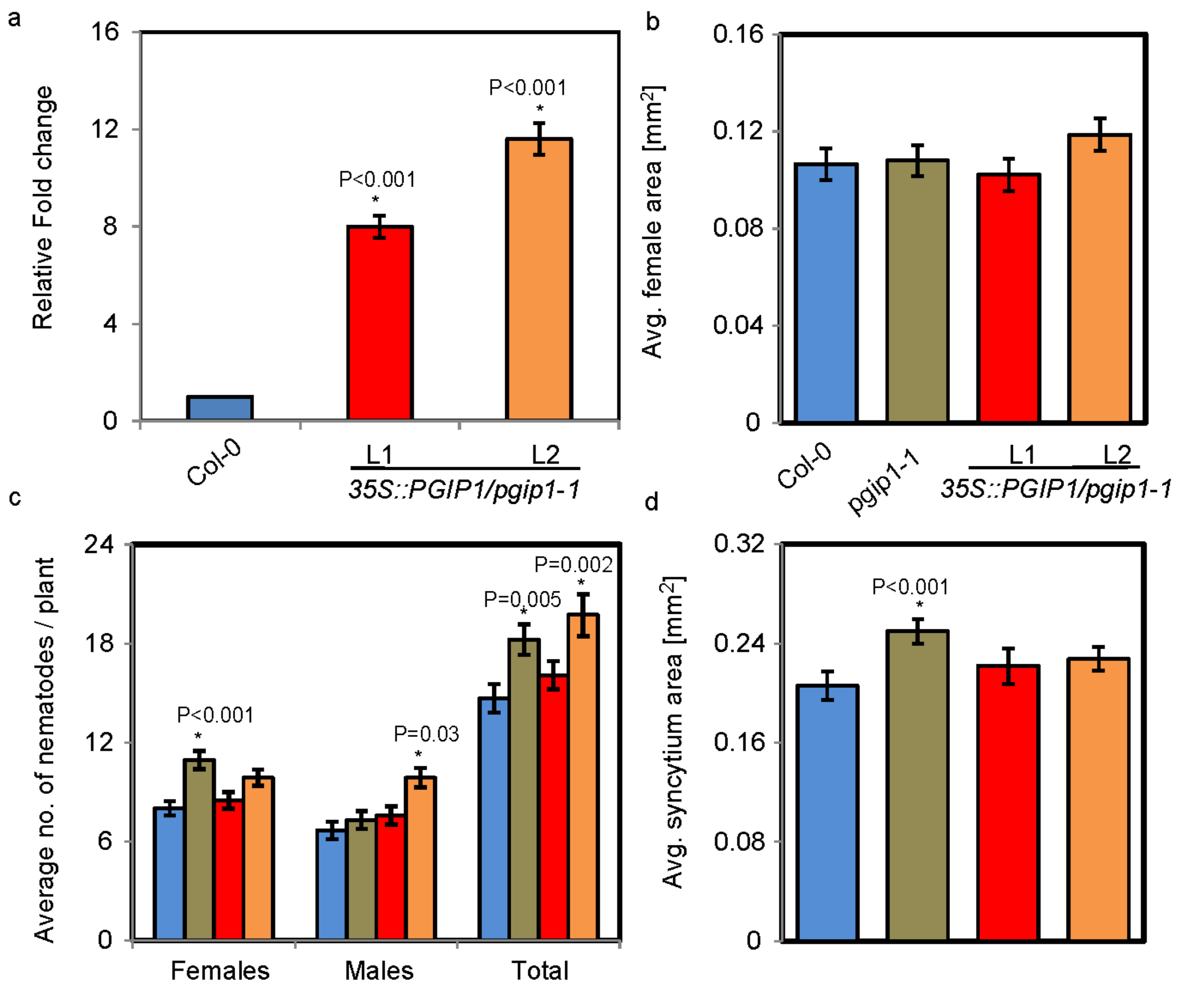

Fig. S6: Cyst nematode infection assays in *PGIP2* overexpression lines. (a) Two independent homozygous lines (L1, L2) overexpressing PGIP2 (*35S::PGIP2*) were selected and analysed for changes in transcript abundance of PGIP. *UBQ5* and *β-tubulin* were used as housekeeping genes to normalize the data. The values represent relative fold change in roots of transgenic lines with the value in uninfected Col-0 root set to one. (b) Average number of females and males per plant present in Col-0 and *PGIP2* overexpression lines at 12 dai. (c, d) Average sizes of female nematodes (b) and plant syncytia (d) in Col-0 and *PGIP2* overexpression lines at 14 dai. (b-d) Bars represent mean ± SE for three independent experiments. Data were analysed using student's T-test (p < 0.05). Asterisks represent statistically significant difference to corresponding Col-0.

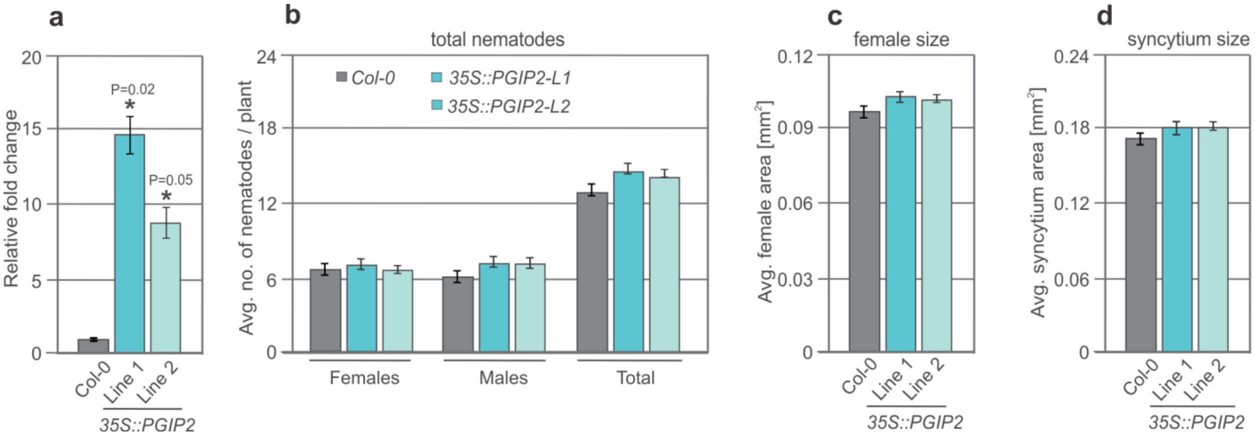

Fig. S7: **Root-knot nematode infection assays in *PGIP1* and *PGIP2* overexpression lines**. (a) Average number of galls per plant present in Col-0, and *PGIP* overexpression lines at 21 dai. (b) Average size of galls per plant present in Col-0 *PGIP* overexpression lines at 21 dai. Data were analysed using student's T-test ($P < 0.05$).

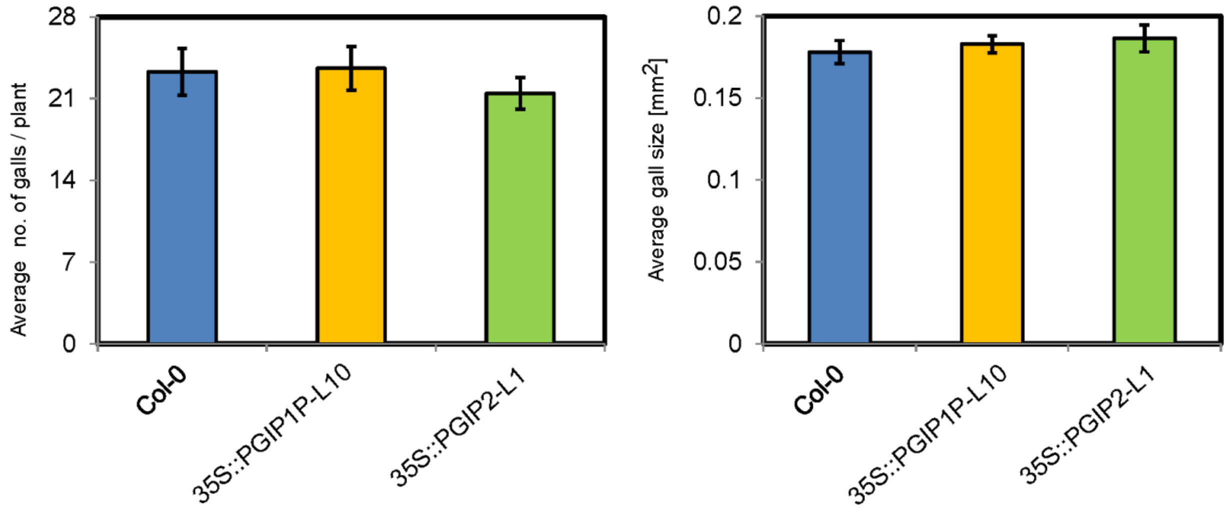

Fig. 8: Cyst nematode infection assays upon OG treatment. (a) Average number of females and males per plant present in water- or OG-treated Col-0 plants at 12 dai. (b, c) Average area of female nematodes (b) and plant syncytia (c) in water- or OG-treated Col-0 plants at 14 dai. (a-c) Bars represent mean ± SE for three independent experiments. All data were analysed using student's T-test (p < 0.05). Asterisks represent statistically significant difference to corresponding H_2O treated Col-0.

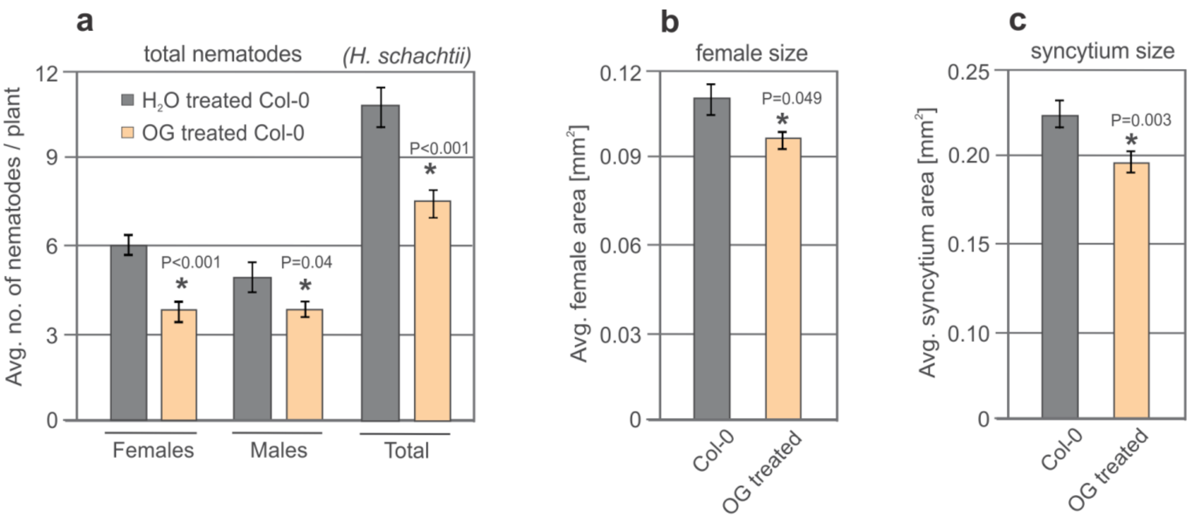

Table S1 Primer sequences used in this work.

Gene	Locus	Forward Primer	Reverse Primer	Efficiency
**UBQ5**	At3g62250	GTTAAGCTCGCTGTTCTTCAGT	TCAAGCTTCAACTCCTTCTTTC	98
**JAZ8**	At1g30135	TGTGTTTTTCTTCAGATGTTACCC	TCTCTGCTTGCGATCGATATT	98
**PROPEP1**	At5g64900	ACGAAGCGAAGAAAGTCACC	TTCGGCTGTTTCGAAGTTCC	102
**NPR2**	At4g26120	AAACCGAGTTGCACTTGCTC	AGTGATGTCCGCTTTTCACC	106
**PAD3**	At3g26830	TTAAGCTCGTGGTCAAGGAGAC	GACCCATCGCATAAACGTTGAC	106
**CYP81F2**	At5g57220	ATCGTGCTAGTGAACGCTTG	TTCGTCCGTTACCAAACACC	95
**CYP71A12**	At2g30750	GCTTCTTGAGATCCCTTGCG	GTGATGTGGTGTTTGGTCCC	98
**PGIP1-qPCR**	At5g06860	AGTCCCTGACCTTCGCCTAT	AGCATCACCTTGGAGCTTGT	93
**PGIP2-qPCR**	At5g06870	AACAAGCTTCAAGGCGATGC	AACCTTGGAGAGATCGAACTGG	95
**PGIP-RTPCR**	At5g06860	CTGACAGGTCCAATTCCTGAC	AATCCATCAAATAAAACATTTTGAA	
**PGIP2-RTPCR**	At5g06870	TCTTGTCCACTCTCCTCCTCA	CCGGAATACTCCCTGTGATG	
**GWpPGIP1:GUS**	At5g06860	GGGGACAAGTTTGTACAAAAAAGCA<br>GGCTGCAAAAGGGCAGGCTAGGCTAA	GGGGACCACTTTGTACAAGAAAGCT<br>GGGTCCTGAGGCAATGTCTTCACCA	
**GWpPGIP2:GUS**	At5g06870	GGGGACAAGTTTGTACAAAAAAGCAGGCTGC<br>ACCAAGCTTATCTCTAGGAT	GGGGACCACTTGACAAGAAAGCT<br>GGGTCGAGTTTTTATGGAAACTATGATTG	
**35S::PGIP1**	At5g06860	ATGGATAAGACAGCGACATTGTGTC	TTACTTGCAAATTTCAAGAGGAGCAC	
**35S::PGIP2**	At5g06870	ATGGATAAGACAATGACACTGTTC	TCACTTGCAACTAGGAAGAGG	
**Left primer**		CTGACAGGTCCAATTCCTGAC		
**Right primer**		TATGAATGCATGGTGCGATAC		
**Border primer**		ATTTTGCCGATTTCGGAAC		

Table S2: An overview of PGIP1 and PGIP2 expression in published and unpublished transcriptomic data. Values are relative fold change. Asterisks indicate significant difference to control.

Locus	Gene Name	Root vs Sync (5+15 dpi) Szakasits et al., 2009	Root vs migratory stage (10 hpi) Mendy et al., (2017)
At5g06860	PGIP1	3.40 *	3.98 *
At5g06870	PGIP2	0.70	1.07
